# A safe anastomotic technique of using the transorally inserted anvil (OrVil™) in Roux-en-Y reconstruction after laparoscopy-assisted total gastrectomy for proximal malignant tumors of the stomach

**DOI:** 10.1186/1477-7819-11-256

**Published:** 2013-10-04

**Authors:** Jian-Wei Xie, Chang-Ming Huang, Chao-Hui Zheng, Ping Li, Jia-Bin Wang, Jian-Xian Lin, Lu Jun

**Affiliations:** 1 Department of Gastric Surgery, Fujian Medical University Union Hospital, No 29 Xinquan Road, Fuzhou 350001, Fujian Province, China

**Keywords:** Laparoscopic surgery, Total gastrectomy, Roux-en-Y, Esophagojejunostomy, Stomach neoplasms

## Abstract

**Background:**

To explore the safety and feasibility of the transorally inserted anvil (OrVil™) in laparoscopy-assisted total gastrectomy for gastric cancer.

**Methods:**

From December 2010 to June 2011, a total of 28 patients underwent laparoscopy-assisted total gastrectomy with a Roux-en-Y-esophagojejunostomy anastomosis with OrVil™. Perioperative treatments, intraoperative data, postoperative complications and hospital length of stay were evaluated.

**Results:**

There were no conversions to the open gastrectomy. The mean operation time was 143 minutes and the mean blood loss was 70 ml. Patients resumed an oral liquid diet on postoperative days 4 to 5. Two patients (7%) who suffered postoperative aspiration pneumonia were cured by conservative treatment. The median hospital length of stay was 9.6 days (8 to 11 days), with no inhospital mortalities. The median follow-up time was 14.8 months (12 to 18 months), and postoperative endoscopic examination revealed no anastomosis stenosis in patients who had dysphagia.

**Conclusion:**

The use of the OrVil™ is technically feasible and relatively safe for Roux-en-Y reconstruction after laparoscopy-assisted total gastrectomy.

## Background

Many studies have demonstrated the advantages of laparoscopy-assisted gastrectomy over conventional open gastrectomy, including reduced surgical invasiveness, less blood loss, less postoperative pain, more retrieved lymph nodes, earlier postoperative recovery, and satisfactory long-term survival [1-3]. Laparoscopy-assisted distal gastrectomy shows a safe and feasible procedure in treating both early gastric cancer (EGC) and advanced gastric cancer, which are both located in the middle and lower third of the stomach [4-6]. For treating proximal EGC, laparoscopy-assisted proximal gastrectomy (LAPG) is an acceptable method except for an increased risk of reflux symptoms [7], while for treating gastric cardia cancer, laparoscopy-assisted total gastrectomy (LATG) becomes more popular than LAPG owing to a lower rate of reflux esophagitis, and it appears to be a rational choice for proximal gastric cancer with deeper invasion [8,9].

However, due to a narrow operating window, there exist some technical difficulties in the course of extracorporeal esophagojejunostomy through minilaparotomy. In obese patients, anvil insertion has become a difficult problem even for the experienced surgeon in esophagojejunostomy after LATG. The difficulty of the anastomosis increases the risk of anastomotic leakage and stenosis. The transorally inserted anvil (OrVil™; Covidien Mansfield, MA, USA) in Roux-en-Y reconstruction is a technically feasible and secure surgical procedure with the advantages of less operation time, easier reconstruction, and acceptable morbidity [10]. The purpose of this study is to assess the feasibility and safety of the use of the OrVil™ for the Roux-en-Y-esophagojejunostomy anastomosis in patients with primary proximal gastric adenocarcinoma.

## Methods

### Patients and materials

From December 2010 to June 2011, 28 consecutive patients with gastric cancer received LATG with a Roux-en-Y-esophagojejunostomy anastomosed by the technique of OrVil™. Criteria for inclusion in the study are: (1) a clear preoperative diagnosis of primary proximal gastric adenocarcinoma should be done; (2) the tumor does not cross the gastroesophageal junction (GEJ) or invade into the distal esophagus within 3 cm above the GEJ; (3) preoperative chest X-ray, abdominal ultrasound and upper abdominal computed tomography scan show no distant metastasis to the liver, lung or abdomen; (4) the radical gastrectomy with D2 lymph node dissection is carried out and pathological diagnosis recommends R0 resection; and (5) the number of lymph node dissection is greater than or equal to 15. Criteria for exclusion from the study are: (1) tumor which invades into the distal esophagus more than 3 cm above the GEJ; (2) T1 or T4b tumors; (3) distant metastases during the operation; (4) a history of upper abdominal surgery; and (5) incomplete information of the pathological diagnosis. The patients were followed up every 3 months by dedicated hospital staff in many different ways, such as outpatient services, personal visits, letters and telephone calls. The routine follow-ups consisted of physical examination, laboratory tests (including carbohydrate antigen (CA)19-9, CA72-4 and carcinoembryonic antigenlevels), chest radiography, abdominopelvic ultrasonography or computed tomography. If dysphagia symptoms were reported, an additional endoscopic examination would be carried out as soon as possible. All of the 28 patients were followed up for 24 to 30 months, and the deadline of follow-up was June 2013.

### Statistical analysis

Statistical analysis was performed using SPSS.v16.0 for Windows (SPSS Inc., Chicago, IL, USA).

### Surgical procedures

#### *Laparoscopy-assisted radical total gastrectomy with D2 lymph node dissection*

The patient was placed in a supine position with legs apart after general anesthesia with tracheal intubation. A 10-mm trocar port for the laparoscope was inserted below the umbilicus, and a 12-mm trocar port was introduced in the left anterior axillary line 2 cm below the costal margin as a major hand port. Then a 5-mm trocar port was inserted in the left mid-clavicular line 2 cm above the umbilicus as an accessory port, and another 5-mm trocar port was placed at the contralateral site. A 5-mm trocar was inserted in the right anterior axillary line 2 cm below the costal margin for exposure. The surgeon stood on the patient’s left side, the assistant on the right, and the camera operator between the patient’s legs. The greater omentum was divided at the mid potion by ultrasonic knife (UltracisionHarmonic Scalpel; Johnson & Johnson, Cincinnati, USA). Then the root of the right gastroepiploic vein and artery were vascularized, and clamped with its origin cut. After opening the pancreatic envelope and separating the membrane of the pancreas, the posterior pancreas space at the superior border of the pancreas was reached and the spleen vascular trunk was revealed. Taking the explored vascular trunk as a starting point, we exposed the branches of the celiac artery and cleaned thoroughly the surrounding fat, connective tissue and lymph nodes of the corresponding vessels. The surgical techniques of the spleen preserving splenic hilar lymph node dissection was similar to the way described by Lu and colleagues [11]. Total gastrectomy with regional lymph node dissection (D2) was performed laparoscopically. After dissection of the gastric lymph nodes, the duodenum and esophagus were cut off by an automated stapler inserted through a trocar of the left abdomen.

#### *Digestive tract reconstruction*

**Figure 1:**
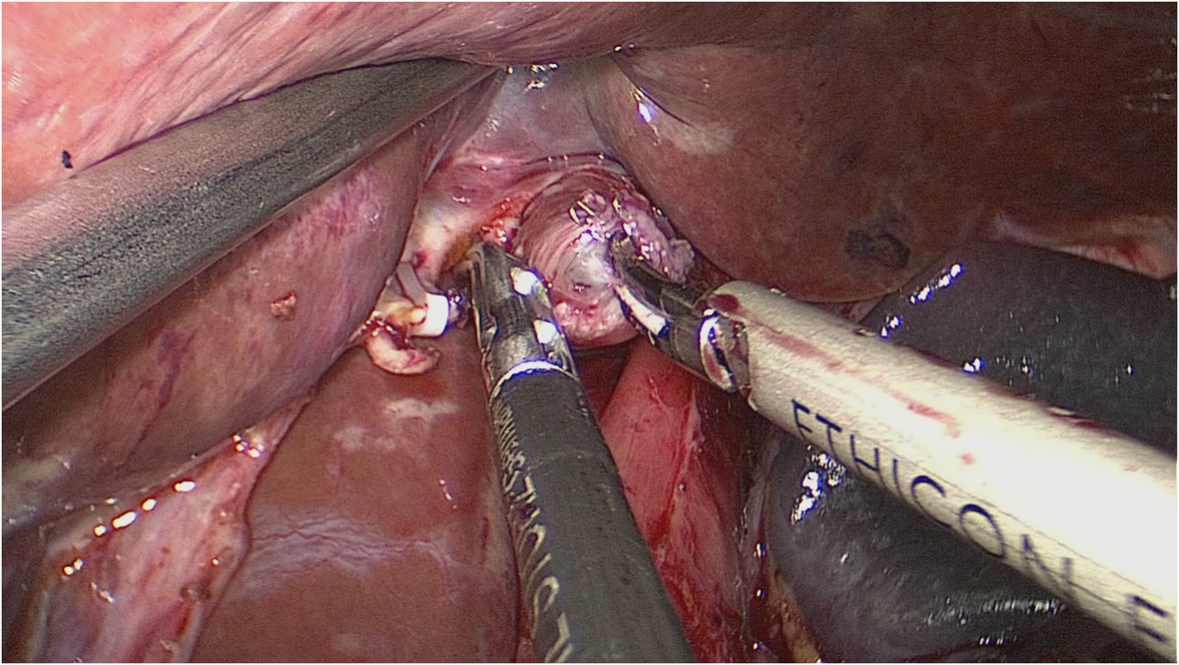
A small hole was created by ultrasonic knife on the corresponding position in the stapled esophageal stump.

**Figure 2:**
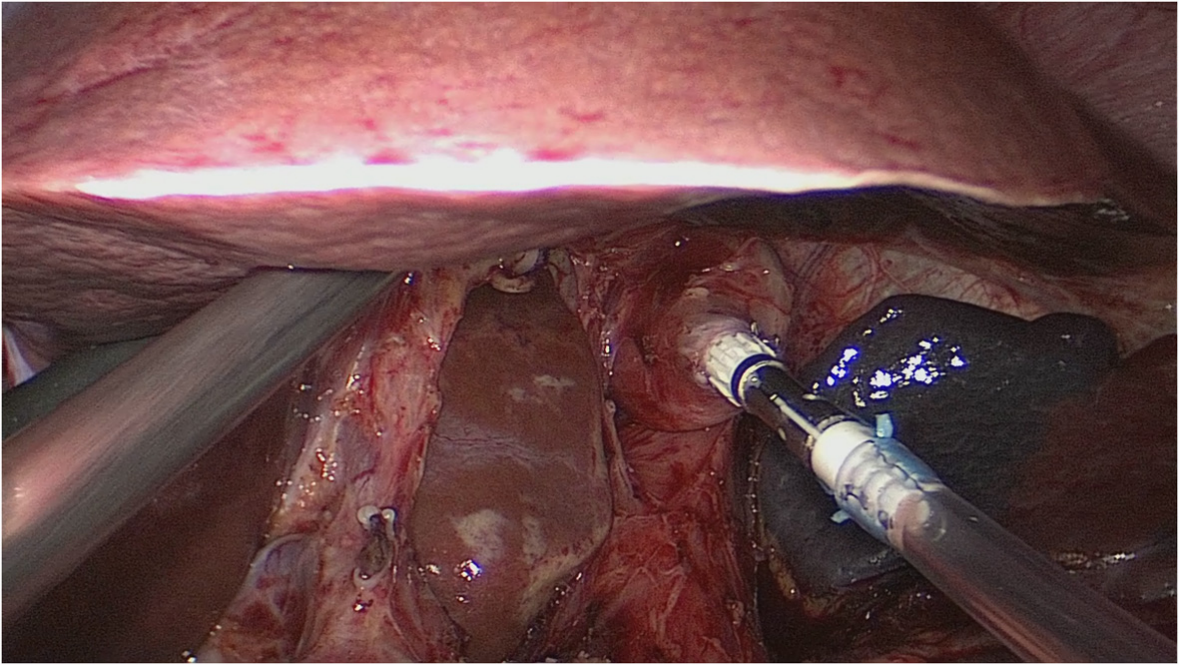
The connecting thread was cut and the orogastric tube would be disconnected from the anvil at once.

**Figure 3:**
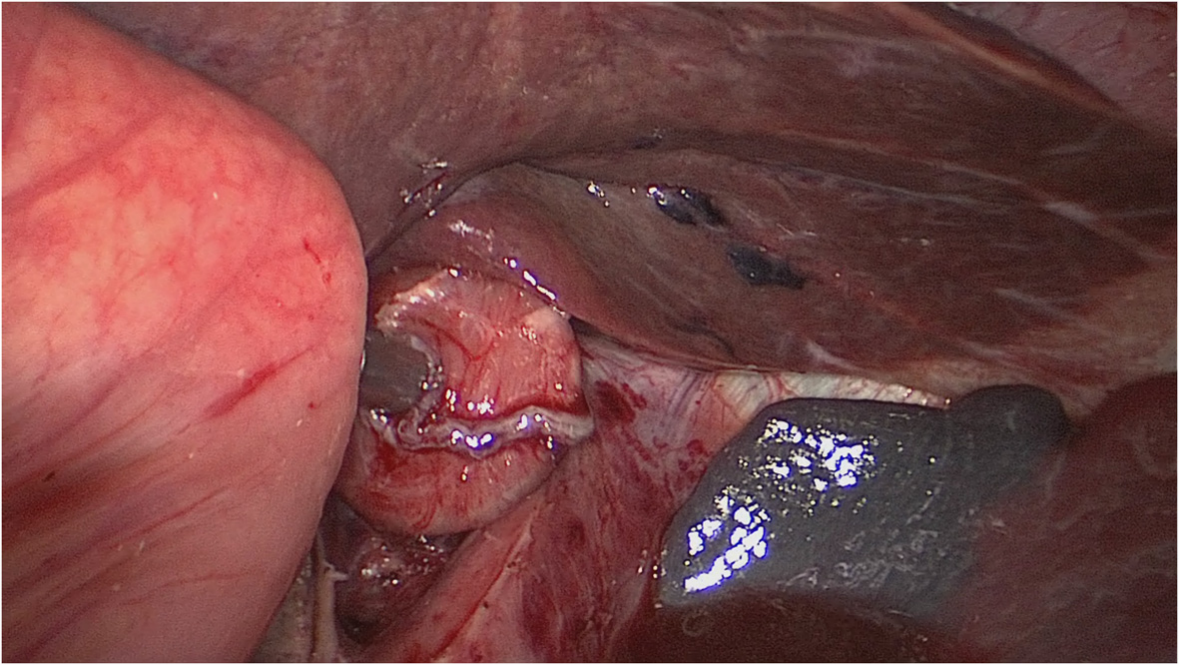
**The main unit of the automated stapler was inserted from the open end of the lifted jejunum to pass through a trocar.** The main unit of the automated stapler and the lifted jejunum were fastened with silk thread. The lifted jejunum was again led within the abdominal cavity.

**Figure 4:**
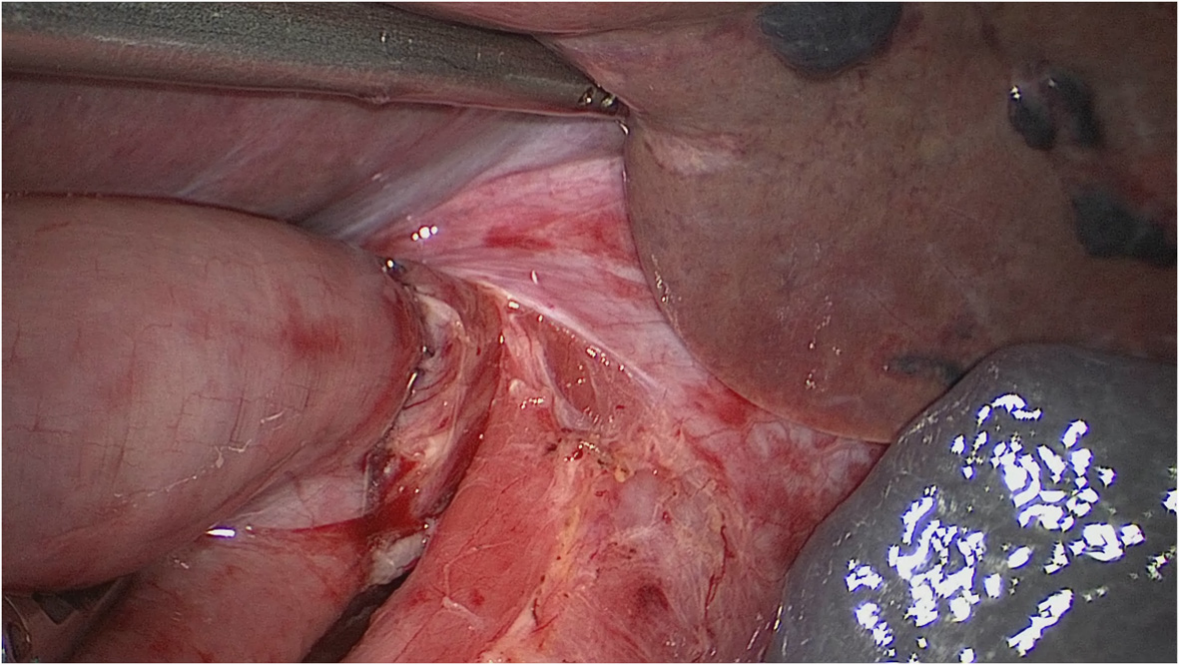
**The anastomosis site was checked in the right direction to make sure there was good blood supply of the esophageal and tension**-**free anastomotic stoma.**

**Figure 5:**
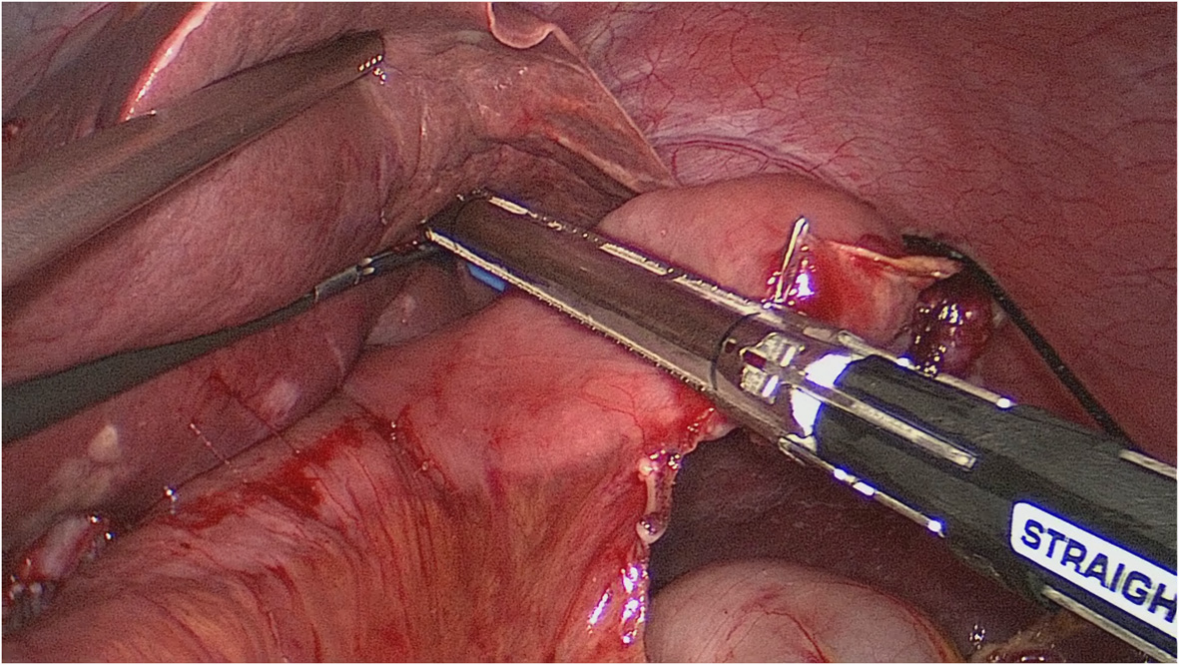
The open end of the lifted jejunum was closed with an automated stapler inserted through a trocar of the left abdomen.

The OrVil™ anvil was passed transorally by the experienced anesthetist through the larynx to the stapled esophageal stump. A small hole was created by the ultrasonic knife on the corresponding position in the stapled esophageal stump, and the tube was pulled out into the abdominal cavity through the hole until the white plastic rubber ring was fully revealed. The connecting thread was cut and the orogastric tube was disconnected from the anvil, then the spike was connected to the esophageal anvil to create an esophagojejunal anastomosis. In the process of removing the tube from the major hand port, particular attention should be paid to keeping the tube from touching abdominal organs or tissues to prevent contamination of the abdominal cavity (Figures 1 and 2). A 3-cm longitudinal minilaparotomy incision was then made at the midline slightly caudal from the ensiform process of the epigastric region. After a wound protector was placed, the stomach was delivered out through the incision for pathological examination. The jejunum was cut off at 15 cm away from the ligament of Treitz and the stump of proximal jejunum was sutured. A 25-mm circular stapler was inserted into the distal limb of the jejunum and was introduced into the abdominal cavity after a second pneumoperitoneum. The anvil and circular stapler were connected and end-to-side esophageal jejunostomy anastomosis was conducted under direct laparoscopic view (Figures 3 and 4). The stump of distal jejunum was intracorporeally sutured by Endo-GIA (Covidien, Mansfield, MA, USA). Finally, a side-to-side jejunojejunostomy was conducted to create a 45-cm Roux-en-Y limb (Figure 5). Abdominal irrigation was performed. A drainage tube was placed through the right upper port site around the esopagojejunal anastomosis and another was placed through the left upper port site around the splenic fossa. The operation was completed by the closure of all wounds.

### Ethical approval

Ethics committee of Fujian union hospital approved this retrospective study. Written consent was given by the patients for their information to be stored in the hospital database and used for research.

## Results

A total of 28 patients (mean age 55 years, range 29 to 69 years) who underwent LATG with Roux-en-Y reconstruction used this method between December 2010 and June 2011. Demographic data, clinical characteristics and pathologic staging are shown in Table 1. The average operation time was 143 minutes (125 to 165 minutes) and estimated median blood loss was 70 ml (65 to 90 ml). Proximal and distal margins were negative in all patients. A median of 36 lymph nodes (23 to 65) were dissected from each specimen, with a median of 1 (0 to 5) histologically positive nodes. No intra-operative technical failures of the anastomosis or deaths occurred. Two patients (7%) who suffered a postoperative aspiration pneumonia were cured by conservative treatment. The median hospital stay was 9.6 days (8 to 11 days) including the two with postoperative complications. All 28 patients were alive without recurrence (100%) with a median follow-up of 28.6 months (24 to 30 months). A symptom of dysphagia occurred in 1 (50%) of 2 patients when the 21-mm EEA (end to end autosuture circular stapler, Covidien, Mansfield, MA, USA) stapler was used and in 3 (12%) of 26 patients when the 25-mm EEA stapler was used. It was suggested that all patients take an endoscopic examination as soon as possible, but the evidence of anastomotic stenosis has not been detected. Table 2 summarizes the results.

**Table 1:** Patient characteristics

Age (years), mean±SD (range)	55±9.5 (29–69)
Gender (male: female)	19:9
Tumor histology (n, %)	
Differentiated	19 (68%)
Undifferentiated	9 (32%)
Tumor location (n, %)	
Gastroesophageal junction	21 (75%)
Fundus	7 (25%)
Pathological stage AJCC classification	
I	10 (35.7%)
II	13 (46.4%)
III	5 (17.9%)
IV	0 (0%)

AJCC, American Joint Committee On Cancer.

**Table 2:** **Operative**/**perioperative data**

Mean operation time (minutes)	143 (125–165)
Mean blood loss (ml)	70 (65–90)
Conversion to open	0
Time to first flatus (days)	2.5 (2.2–2.9)
Time to resumption of oral intake (days)	4.1 (3.0–5.0)
Mean number of lymph nodes	36.4 (23–65)
Mean number of positive nodes	1 (0–5)
Median length of stay	9.6 (8–11)

## Discussion

The means of gastrointestinal tract reconstruction after LATG still remain controversial [12-16]. Because there are less postoperative complications and reflux after the total gastrectomy, and because there is better nutritional status and the ideal body weight can be maintained, the Roux-en-Y esophagojejunal anastomosis after LATG has been widely used, with the features of ease of use and speed [17,18]. Small incision-assisted reconstruction of the digestive tract is a method which drags the required intestines outside the abdominal cavity, and then completes the reconstruction extracorporeally or places stapling through the incision. It reduces the operation fee significantly, and the anastomosis is safe and reliable, which makes it widely used nowadays. However, due to visual restrictions, it is difficult to complete anastomosis in obese patients or men with a deep abdominal extent using small incisions. The placement of the stapler anvil is one of the limiting features of LATG. The technique of using the OrVil™ device for LATG anastomosis was first described by Jeong and Park in 2009 [19]. It successfully overcame the difficulties of anvil placement, and was fast and reliable [19]. More recently, Kunisaki and colleagues [10] reported the short-term clinical efficacy of gastric cancer using conventional digestive tract reconstruction after LATG compared with that after the OrVil™-assisted reconstruction, which revealed the average operation time and blood loss of the latter to be 64.5 minutes and 68.9 ml, respectively. Our data show a similar average operation time and average blood loss. Compared with the conventional laparoscopic-assisted reconstruction using small incisions, the application of the OrVil™ device reduces the difficulty of reconstruction of the digestive tract, and shortens the operation time and postoperative hospital stay [20]. Therefore, it is feasible to reconstruct the digestive tract with the use of the OrVil™ device.

In spite of the shortened operation time using the OrVil™ device, the digestive tract reconstruction-related complications still remain. Esophagojejunal anastomotic leak and stenosis are associated with considerable mortality. We reported an acceptable level of complications, and only 2 of the 28 patients suffered pulmonary infection; the causes might include long-term smoking and general poor health. Reported leak and stenosis rates of end-to-side circular staple anastomosis with OrVil™ after LATG ranged from 0 to 16.7% and from 0 to 33.3%, respectively (Table 3). This study showed no occurrence of anastomotic leak and stenosis except for four cases with a symptom of dysphagia. The reasons for these cases might be: cutting off the lower esophageal before anastomosis;operating *in situ* avoided mucosal injury by excessive traction on the esophagus; and reduced risks of anastomotic leakage. We had a broader vision to conduct esophagojejunal anastomosis under the direct sight of a laparoscope so that we can observe the stoma from many angles and find the weakness in order to repair them in time. As far as possible, we used a 25-mm EEA stapler in most cases because the use of the 21-mm DST-EEA causes anastomotic stenosis at a higher rate [21].

**Table 3:** **Relevant reported results of end**-**to**-**side circular staple anastomosis with OrVil**™

**Author**	**Year**	**Gastrectomy technique**	**Number**	**BMI (kg/m**^**2**^**)**	**Mortality (%)**	**EJ leak rate (%)**	**EJ stenosis rate (%)**
Jeong and Park [19].	2009	TLTG	16	23.0^a^	0	0	0
Sakuramoto *et al.*[22].	2010	LATG	26	24.0^b^	0	0	3.8
Kachikwu *et al.*[23].	2011	TLTG	16	24.9^a^	0	0	18.8
Kunisaki *et al.*[10].	2011	LATG	30	23.0^b^	3.3	3.3	N/R
Marangoni *et al.*[24].	2012	TLTG	13	N/R	7.7	0	N/R
Shim *et al.*[25].	2012	TLTG	12	24.3^b^	0	16.7	33.3
Liao *et al.*[26].	2013	LATG	19	21.2^b^	0	0.5	0.5
Zuiki *et al.*[21].	2013	LATG	52	21.6^b^	0	1.9	21
Chong-Wei *et al.*[27].	2013	TLTG	16	N/R	0	0	N/R
Lafemina *et al.*[28].	2013	TLTG	17	27.1^a^	0	5.9	5.9

^a^Median.^b^Mean. BMI, body mass index; EJ, esophagojejunal; N/R, not reported; LATG, laparoscopically assisted total gastrectomy; TLTG, totally laparoscopic total gastrectomy.

Furthermore, because of the closure of the lower esophagus, the abdominal cavity pollution caused by digestive juicescan be avoided, which reduces the incidence of abdominal infection. In addition, without insertion of the anvil through the abdominal cavity, purse-string suture of the esophageal stump offers more convenience for anastomosis and a higher cut site which ensures enough proximal incisal margin. In the 28 patients in which gastric cancer was located in the cardia and fundus, the tumors were completely resected and negative margins were acquired. There was no recurrence and metastasis in these cases through follow-up. Therefore, we believe that it is safe to reconstruct the digestive tract after LATG by using the OrVil™ device because it does not increase the risk of surgical complications and recurrence.

We realize that we should pay attention to the following points during the application of the OrVil™ device to assist digestive tract reconstruction after total gastrectomy. (1) The insertion of the OrVil™ catheter stapling anvil requires the cooperation of an experienced anesthesiologist who is familiar with esophageal anatomy. Sufficient lubrication is necessary before placement, and it must be confirmed that the spherical surface of the anvil faces the direction of the patient’s palate when inserting the catheter. With the help of the laryngoscope and by lifting the back of the neck we can make the lower jaw close to the sternum, or evacuate the gasbag of the endotracheal tube to facilitate the passage of the anvil into the esophagus. The entire placement process should be gentle and slow, and force should not be used in case of resistance to prevent damage to the esophageal lining [29]. (2) The contents of the stomach and esophagus should be drained before esophageal transection and the abdominal organs should not be touched during the retrieval of the stomach tube. The main operating bore trocar should be replaced to prevent abdominal infection. (3) The stapler puncture needle should be parallel to the center of the anvil rod. The clip applier can be replaced by a Hem-o-lok clip applier to clamp the anvil center rod in order to increase the power of grip. A click on correct matching can be heard and felt, while the orange circle on the stapling must be completely covered. (4) The direction of the distal jejunal loop should be made clear in order to avoid Roux jejunal loop reversing. Excessive pushing of the distal intestine should be avoided during the matching of the puncture needle and the anvil center rod in order to prevent anastomosis failure caused by intestinal damage. The esophageal stump after anastomosis may not be completely embedded within the stoma, but this does not influence the anastomotic quality and there is no need to embed the stoma once again.

## Conclusions

Application of the transoral anvil delivery system in Roux-en-Y reconstruction is a feasible surgical procedure for LATG. With careful manipulation and proficient operation skills, the procedure can be performed safely with acceptable morbidity and mortality.

### Consent

Written informed consent was obtained from the patient for the publication of this report and any accompanying images.

## Abbreviations

CA: Carbohydrate antigen; EEA: End to end autosuture circular stapler; EGC: Early gastric cancer; GEJ: Gastroesophageal junction; LAPG: Laparoscopy-assisted proximal gastrectomy; LATG: Laparoscopy-assisted total gastrectomy.

## Competing interests

The authors declare that they have no competing interests.

## Authors’ contributions

JWX and CMH conceived the study, analyzed the data and drafted the manuscript. CHZ helped revise the manuscript critically for important intellectual content. PL, JBW, JXL and LJ helped collect data and design the study. All authors read and approved the final manuscript.
